# The Future of Food

**DOI:** 10.3390/foods13040506

**Published:** 2024-02-06

**Authors:** Charis M. Galanakis

**Affiliations:** 1 Research & Innovation Department, Galanakis Laboratories, 73131 Chania, Greece; cgalanakis@chemlab.gr; 2 College of Science, Taif University, Taif 26571, Saudi Arabia; 3 Food Waste Recovery Group, ISEKI Food Association, 1190 Vienna, Austria

**Keywords:** food sustainability, food security, protein, food waste, bioeconomy, biotechnology

## Abstract

The global food systems face significant challenges driven by population growth, climate change, geopolitical conflicts, crises, and evolving consumer preferences. Intending to address these challenges, optimizing food production, adopting sustainable practices, and developing technological advancements are essential while ensuring the safety and public acceptance of innovations. This review explores the complex aspects of the future of food, encompassing sustainable food production, food security, climate-resilient and digitalized food supply chain, alternative protein sources, food processing, and food technology, the impact of biotechnology, cultural diversity and culinary trends, consumer health and personalized nutrition, and food production within the circular bioeconomy. The article offers a holistic perspective on the evolving food industry characterized by innovation, adaptability, and a shared commitment to global food system resilience. Achieving sustainable, nutritious, and environmentally friendly food production in the future involves comprehensive changes in various aspects of the food supply chain, including innovative farming practices, evolving food processing technologies, and Industry 4.0 applications, as well as approaches that redefine how we consume food.

## 1. Introduction

The global food scene is undergoing profound difficulties driven by complex factors, encompassing population growth, climate change, a condensed period of multiple crises (e.g., wars, geopolitical conflicts, and pandemic-related implications), and evolving consumer preferences [1,2,3]. For instance, the world’s population is predicted to reach an estimated 9.7 billion by 2050 [4], pressing resources management and placing an increasingly urgent demand on producing safe, nutritious, and sustainable food. The rapid increase in the global food demand is accompanied by urbanization, a corresponding increase in environmental impacts, and agricultural expansion, which necessitates the sustainability and resilience of our food systems [5,6].

In this era of challenges and opportunities, discussions surrounding the future of food have risen in both the scientific community and societies. Thus, it is critical to optimize production by enhancing yields on underperforming lands, increasing crop efficiency, encouraging dietary shifts, and minimizing food wastage, all while mitigating environmental impacts [7]. Likewise, the food sector needs a transformation that aligns with the shift from fossil fuels to bio-based products and a climate-neutral economy, digitalization, Industry 4.0 technologies in food logistics, and bioresource valorization [3,8,9]. Priorities and governmental policies should include agroecological practices (e.g., rooftop agriculture, vertical farming, and precision agriculture enabled by IoT applications) and the “blue bioeconomy” (e.g., through aquaculture and multitrophic systems) [10], food safety and digital traceability [11], equitable food distribution [12], livestock farming integrated into crop–livestock systems and adhering to “One Health” principles [13,14], and shorter supply chains, with the approaches that redefine the way we consume food (e.g., plant-based meat substitutes and lab-grown meat) playing a pivotal role [2].

Clear scientific targets such as a shift to healthy diets and sustainable consumption (reduction in red meat and sugar) [14,15], nutritious and functional food production, bioactive-rich foods [16], and tailoring food choices based on personalized diets and the role of nutrigenomics [17], together with the role of cultural diversity in shaping culinary innovations [18], are crucial to guide this transformation. However, other promising innovations, like the improvement in crops through Clustered Regularly Interspaced Short Palindromic Repeats and CRISPR-associated protein 9 (CRISPR-Cas9) gene editing [19], may raise concerns regarding safety and public perception. Subsequently, consumer preferences should not be underestimated but used as guidance for developing future foods.

**Figure 1:**
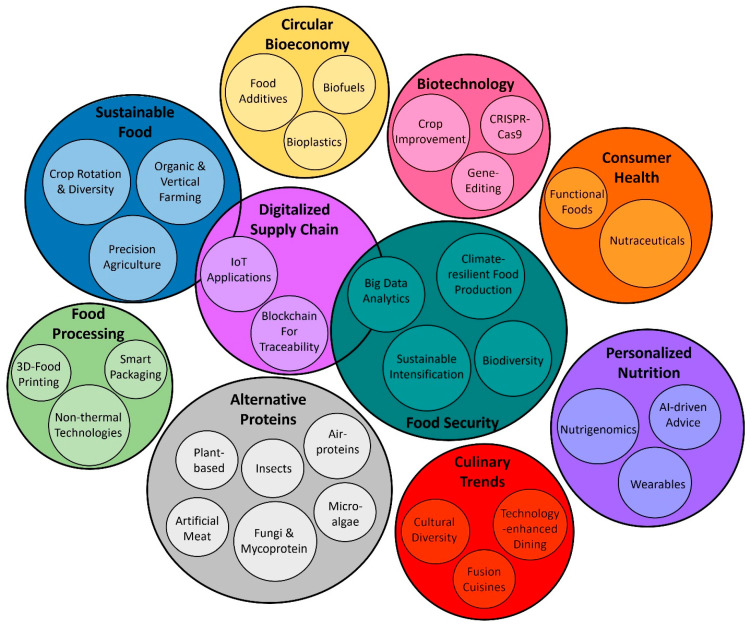
Illustration of the dimensions that constitute the future of food.

This review article examines the multifaceted dimensions that collectively constitute the future of food (Figure 1). Starting from critical aspects that encompass sustainable food production, alternative protein sources, culinary innovations, the impact of biotechnology, health-related trends, and the digitalization of the food chain, the review delves into food safety, security, and resilience strategies and the role of circular bioeconomy in sustainable food systems. By weaving together insights from these diverse domains and web of factors, the goal is to provide food researchers with a holistic view of the dynamic trajectory the food industry is embarking upon. The latter should be defined by innovation, adaptability, and a shared commitment to a resilient and nourishing global food system.

The review is part of the research performed in the SecureFood project and deliverable D2.1 scheduled for November 2024 of the SecureFood Project.

## 2. Sustainable Food Production

To adequately feed the projected global population of 10 billion by 2050, global food production should increase by at least 70% [20,21]. However, the old practice of simply growing food production without enhancing the efficiency of the food systems belongs to the past. The rising demand for processed foods coupled with the depletion of resources has led the industry to increase its attention towards sustainability. Achieving sustainability is a complex endeavor that requires changes in food consumption patterns, including reducing the link between income and animal product consumption, updating existing farming models, investing in climate-resilient agricultural systems, and proactive policies for structural development in agriculture and food trade regulation [22]. It also necessitates reconsidering agricultural practices, reducing food loss and waste, maximizing the conversion of raw materials into consumer products, and integrating and collaborating activities across all stages from farm to fork [3,23,24].

Certain practices, like reduced tillage, organic farming, and drip irrigation, are already well-incorporated, but biofertilizers, crop rotations, intercropping, and agroforestry are less integrated [22]. These practices have numerous advantages and should be adapted to achieve sustainable food systems. For instance, the innovative approach of diversifying and intensifying crop rotations under no-tillage management is known to boost crop yield [25] and agricultural profitability simultaneously [26,27], while decreasing the environmental impact [28]. This is more evident under tropical and subtropical conditions, particularly in crops like soybean, maize, and wheat, where diversified rotations outperform double cropping in profitability and gross margin [29]. Compared to a continuous monoculture, diversified crop rotations improve pest and disease control, enhance soil fertility [30], and mitigate adverse effects on production systems’ performance from climatic variations and biotic parameters, such as weeds and diseases [31].

Organic farming is another approach that can boost soil health, improving nutrient availability and crop quality and avoiding chemical inputs (e.g., fertilizers, pesticides, growth hormones, and livestock feed additives) [32,33]. Improved soil fertility, biodiversity conservation, carbon sequestration, and climate change mitigation can be achieved by promoting agroforestry, which involves intentionally fusing woody elements with lower-level agricultural production [34,35]. Additionally, agriculture needs an urgent re-evaluation of vertical farming, which is designed to optimize crop yield in confined spaces (e.g., cultivating crops in vertical stacks within controlled indoor environments and utilizing hydroponic or aeroponic systems) with shorter growth cycles, reduced water consumption, and pesticide-free food production. This practice benefits urban areas and densely populated regions by securing a consistent supply of fresh, nutritious produce and enhancing food system resilience [36]. Additionally, urban agriculture (e.g., rooftop cultivation and indoor farming) integrates water and energy resources into evolving urban food systems, aligning with the needs of smart and sustainable cities [37].

Aiming to redefine future agriculture, Industry 4.0 applications, such as Artificial Intelligence (AI), Internet of Things (IoT), and cyber–physical systems, are transforming agricultural productivity by facilitating monitoring, precision forecasting, and providing comprehensive insights and system optimization [2,3]. For instance, AI, particularly machine learning, is crucial to process large datasets for crop monitoring. These advancements have streamlined data storage and processing, replacing outdated methods [38]. Precision agriculture encompasses the use of several technologies (e.g., soil sensors, mapping to satellite-based positioning and linked devices, remote control tools, and automated steering) for more efficient operations, reduced resource inputs, and enhanced profitability to provide farmers with more control over their fields and teams [39]. However, real-time IoT sensor monitoring improves decision-making efficiency, and its adoption must address security, complexity, privacy, and reliability concerns [40]. With ancillary technologies like IoT, unmanned aerial vehicles, field robots, and herbicides, AI revolutionizes weed control for holistic, data-driven approaches, reducing herbicide usage and fostering sustainability [41]. On the other hand, digital twins (DTs) offer great potential, allowing experts and farmers to simulate scenarios, test strategies, and predict outcomes with precision. This innovation revolutionizes crop management, optimizing resource usage, reducing environmental impact, and enhancing crop yields [42].

## 3. Digitalization of the Food Supply Chain

The traditional food supply chains face multiple issues, including food safety, fruit and vegetable losses, disruptions due to global events, inadequate labeling, and production delays [2,6]. Food safety and quality assurance challenges include detecting alterations in harmful substances and ensuring authenticity accuracy in cultivation, variety, and geographical origin [43]. The future of food labeling is expected to include more detailed and transparent information about ingredients, sourcing, and sustainability, catering to consumers’ desire for a greater awareness of the products they purchase. For instance, digital and QR-code-based labeling systems may also become more prevalent, offering consumers instant access to comprehensive product information via their smartphones. Likewise, challenges like data exchange, material flow control, economic obstacles, and food waste highlight the need for a further digitalization of the supply chain from farm to fork using tools such as information technologies (Bluetooth, IoT and Distributed Ledger Technology (DLT)), sensors (Wireless Sensor Network (WSN), Temperature and Thermal Imaging (TTI), Barcode, and Radio-Frequency Identification (RFID)), location-based tech (Remote Sensing (RS), Global Positioning System (GPS), and Real-Time Location System (RTLS)), and web apps for the real-time monitoring and tracking of food products [44,45]. DLTs enhance transparency and trust among supply chain participants while maintaining security and privacy, whereas DTs improve the precision of digital representations, enabling process simulation and optimization [46]. IoT solutions also reduce waste, enhance efficiency, and improve visibility in logistics processes [40,47]. Food supply chains rely heavily on collecting and analyzing information for product tracking and stakeholder demands to enhance efficiency, reduce costs, benefit producers, minimize environmental impacts, and support fresher and healthier food transportation [46]. Subsequently, future food quality is expected to be signaled through the above technologies, providing consumers with real-time access to information about the origin, production methods, and safety of food products. However, other challenges must be addressed, such as sustainability concerns, government regulations, and integrating privacy preservation with food traceability [48].

Blockchain technology holds significant potential to enhance transparency and consumer trust in the food supply chain. Its decentralized nature ensures data integrity, making it resistant to manipulation. This technology assigns unique digital identifiers to food products, enabling traceability and preventing fraud, even identifying foodborne illnesses. The advantages include data sharing within the industry, immutability, reduced fraud risks, shorter transaction times, and lower costs [49]. The extensive traceability is facilitated through a common technological platform, allowing consumers to access product details via smartphones and offering crucial information on origins, batches, processing, and expiry dates.

Nevertheless, implementing blockchain in supply chains can be complex (e.g., lacking standardized procedures), demanding stakeholder collaboration. Additional research and universal evaluation models are required for seamless integration with the IoT, addressing issues such as food adulteration and authentication [50]. Furthermore, when integrated with emerging technologies, like AI, big data analytics, RFIDs, NFC, IoTs, cloud computing, and analytical methods for the detection of foodborne pathogens, blockchain can further digitalize food supply chains, enhancing management, automation, efficiency, sustainability, and verifiability [51]. This integration holds transformative promise for the food sector, particularly retailers that urge suppliers to embrace blockchain to enhance supply chain security and minimize food risks [43]. Retail formats that are expected to expand include e-commerce, driven by convenience and personalization, as well as sustainable and ethical retail emphasizing eco-friendly practices. Technology-driven stores and direct-to-consumer brands also represent growth areas in the retail industry [42,43,44,45,46,50,51,52,53].

On the other hand, using AI concepts, machines, and deep learning methods has become increasingly prevalent in studies aimed at analyzing vast food databases to explore aspects like flavors, food compositions, and chemical compounds [52]. Additionally, sensory analysis and taste testing conducted by AI systems may play a role in assessing food quality and tailoring recommendations to individual preferences. Indeed, an increase in personalized marketing communications driven by data analytics and consumer preferences is anticipated in food marketing. Online shopping for groceries is also expected to grow in this direction, focusing on improving user experiences and incorporating AI-driven recommendations to enhance the shopping process. Consequently, many food companies utilize AI and big data to enhance product quality, cater to consumer demands, and drive the industry towards a more intelligent and sustainable future [53]. For example, big data can be used to analyze consumer perceptions after gathering them from social media platforms to aid health food manufacturers in enhancing their products to align with customer preferences [54].

## 4. Food Security and Climate-Resilient Food Supply Chain

Food security entails that everyone consistently has physical, social, and economic access to a sufficient supply of safe and nutritious food, meeting their dietary needs for a healthy life. Food insecurity affects disproportionately low-income communities and individuals with health issues and ranges from household-level worries about accessing food to severe child hunger due to insufficient supply [55]. Food supply chains (as critical infrastructure) and food security are vulnerable to significant disruptions, pandemics, and war conflicts. These crises reveal the need for research on optimizing designs and policies, encouraging operations, and applying big data analytics [2,56]. More specifically, expected developments in food policies include a stronger focus on sustainability and environmental impact, with regulations promoting eco-friendly practices and reduced food waste. Health-conscious policies may also encourage the reformulation of products to reduce salt, sugar, and unhealthy fats, promoting healthier diets while enhancing food safety measures and ensuring transparent labeling to empower consumers in making informed choices. In addition, supply chain resilience in the agri-food sector can be improved by customer-centric decision-making, proximity-based distribution, and cooperative models, providing a competitive advantage during demand fluctuations, as demonstrated during the COVID-19 pandemic [57].

On the other hand, mitigating global warming to limit it to 1.5 °C while dealing with the growing worldwide population and addressing the increasing demands on land for food security and carbon sequestration is a complex issue [58,59]. Climate change and variability pose significant challenges for smallholder farmers in developing countries (e.g., in African and Asian regions), where many depend on farming [60]. Severe and frequent extremes, like droughts, heavy rainfall, temperature shifts, and strong winds, have reduced crop yields of key crops (e.g., maize, rice, and potatoes), negatively affecting food supplies, income, and food security for these farmers [61].

Traditional intensive agricultural systems often rely on heavy resource inputs, monocultures, and heavy chemical use, which pose risks to soil biodiversity and ecosystems. Unsustainable intensification methods have led to soil degradation, including acidification, erosion, salinization, and contamination [62]. The sustainable intensification of production on existing cultivated land has been proposed as a potential solution to balance these demands by enhancing agricultural productivity while mitigating adverse environmental impacts [59]. Although the definition of sustainable intensification has evolved, it primarily entails achieving significant yield increases in underutilized or degraded agricultural areas while revitalizing natural resources. This approach strives to preserve soil biodiversity, reduce nutrient losses, and enhance efficiency. New strategies for sustainable land management and production optimization include lime to counter soil acidification and improve pH, biochar and zeolites for enhanced nutrient supply and water retention, and nitrification inhibitors to reduce N_2_O emissions and NO_3_ leaching [62]. The future of sustainable intensification research is expected to prioritize ecological and social benefits, with increased involvement from developing nations and ongoing cross-regional collaboration [63]. However, the extent to which sustainable intensification can increase yields remains uncertain. Sustainable intensification practices in South Asia yielded an average increase of 21%, with residue retention and organic fertilizers showing notable benefits. Profitability was not always guaranteed, and yield gains were modest compared to the existing yield gaps [58].

Aiming to increase food security, environmental preservation, productivity, and production diversity in a changing climate, smallholder farmers must adopt preparedness strategies to lessen vulnerability and crop losses. These strategies should emphasize access to agricultural inputs, market data, crop diversification, irrigation, and climate-smart tech while accounting for community perspectives [61]. Addressing climate change impacts requires intensified cooperation among research institutions, policymakers, crop managers, and farming communities [60]. Sustainable practices, like climate-smart soil management and drought-resistant crop breeding, will become vital in the following years. Farmer involvement, government policies, and subsidies are crucial for environmentally friendly crop production, limiting annual field movement and mitigating deforestation [21]. Effective policy implementation will require a more holistic approach considering productivity and environmental sustainability in diverse agricultural systems. Additionally, multisectoral collaborations and multilevel interventions are deemed necessary to mobilize resources for addressing the combined impacts of climate change, food insecurity, and the pandemic, especially in lower- and middle-income countries [64].

Moreover, incorporating carbon sequestration and biodiversity into sustainable intensification goals can enhance its relevance and mitigate the focus on productivity and yields [59]. Though under threat, biodiversity plays a pivotal role in mitigating climate change by serving as a natural defense mechanism against extreme climatic events and releasing greenhouse gas (GHG) emissions. Soil biodiversity is critical to ecosystem functions, such as soil aggregation, carbon sequestration, organic matter decomposition, and nutrient cycling [62]. Thus, its preservation is becoming very important for the food sector, and different actions are required in this direction. For instance, Crop Wild Relatives are wild plant species closely related to cultivated crops and possess valuable genetic traits that enhance crop productivity and resilience in changing environmental conditions [60].

## 5. Alternative Protein Sources

Dietary proteins are pivotal in human nutrition and addressing food security challenges. Subsequently, the global protein ingredient market is continuously growing, mirroring the escalating demand and the depletion of essential resources driven by population growth. Although animal-based proteins possess a substantial nutritional quality, increased meat consumption is associated with ethical challenges and significant environmental concerns due to increased GHG emissions contributing to climate change [65]. On the other hand, diets that include higher amounts of alternative proteins and lower meat consumption are increasingly being recognized for their positive impacts on the environment and human health, e.g., the consumption of plant proteins is associated with a reduced chronic disease risk [66,67].

In response to these challenges, researchers actively pursue sustainable protein sources that yield nutritional and economically feasible products. This is not always an easy task since animal proteins are regarded as complete due to their content of vital amino acids, a characteristic not shared by all plant proteins, which are often deemed incomplete due to their deficiency in certain amino acids (e.g., lysine, threonine, and sulfur-containing amino acids like cysteine and methionine) required for proper human growth. Certain plant crops, like soybeans, peas, quinoa, and amaranth, contain all the essential amino acids [68]. Other plant-based proteins, like oats, lupin, or wheat, effectively meet amino acid needs when combined with animal proteins. Protein blends can mimic animal-based protein characteristics, and in this sense, connecting plant (e.g., wheat, oat, lupin, and wheat) and animal proteins can guarantee adequate essential amino acid intake [67,68,69]. Further research to enhance nutrition, simulate flavors, and improve protein functionality is necessary to appeal to substitutes for conventional meat products [70].

Cultured meat presents another promising alternative protein source. Research is now directed toward the sustainability of lab-grown meat production and consumer acceptance, as the success of these products relies on replicating meat’s taste. Consumer preferences concerning different meat varieties and scalability in developing countries and regions characterized by low meat consumption levels are other significant challenges that should be addressed [71]. Beyond conventional food markets, lab-grown meat could find a market in pet food and various non-food products, reducing poaching [72].

Nevertheless, cultured meat confronts several technical challenges, including replicating meat diversity across animal species, breeds, and cuts. Safety advantages from cultured muscle cells exist but concerns about dysregulation and nutritional composition persist. There is also an ongoing debate about the environmental benefits of GHG emissions, significant competition from plant-based alternatives, and uncertainty surrounding the religious acceptability of cultured meat, whether it aligns with Kosher or Halal dietary requirements. Moreover, despite ongoing research, the future competitiveness of cultured meat against traditional meat and substitutes remains uncertain as policymakers face pressure to regulate cultured meat production [72,73]. Policymakers should use these insights for tailored marketing strategies considering societal and economic factors [71]. Effective communication, scale-up production, real-time sensing for nutrient recycling, and regulatory guidelines are essential aspects to be addressed by policymakers [70].

Edible insects present another sustainable alternative source of protein and other nutrients, like fat and minerals, offering the potential for improving several foodstuff items, including snacks, pasta, protein bars, and bread. Nearly 2000 edible insect species (grasshoppers, bees, caterpillars, crickets, locusts, wasps, beetles, and ants) exist globally, commonly consumed in the tropical and subtropical regions of Africa, Asia, and the Pacific regions, but encounter limited acceptance in Western countries [74]. Insect proteins are nutritionally valuable, with functional properties like gelling, foamability, and emulsification. Additional advantages include the year-round availability and low environmental impact compared to meat proteins [75]. The research into extraction methods (e.g., wet fractionation and non-thermal techniques, like ultrasound and microwave treatments) and bioactive peptides from insects is ongoing, and processes such as utilizing microbial fermentation, simulating gastrointestinal digestion, and applying commercial enzymes are considered for the extraction of antidiabetic, antioxidant, and anti-hypertensive peptides [76]. Nevertheless, the broader development of the insect market faces challenges that need to be surpassed, e.g., defatting methods for boosting insect protein functionality, allergenicity, safety issues, consumer acceptance, willingness to pay, and clear regulations [74,75,77,78]. Collaborating with healthcare experts is recommended, and further measures may be necessary to ensure the production of high-quality prebiotics from insects [79].

Algae’s high protein content and low environmental impact make them another promising protein source to produce supplements and food additives in chocolates, bread, noodles, beverages, and beer, as well as meat analogs with a fibrous texture (using high moisture extrusion) and nutraceutical claims related to bioactive peptides and antioxidant capacity [80,81]. Several extraction methods can efficiently isolate algal proteins for nutritious and low-cost food production, although there is still uncertainty about their bioavailability, necessitating further in vivo research studies. Subsequently, further research is needed to improve protein extraction and purification methods [81], and non-thermal technologies can be used in this direction [82]. For instance, ultrasound-assisted extraction has been used to enhance the yield of antioxidants from various sources, including microalgae, offering shorter extraction times, reduced solvent consumption, and lower temperatures [83].

Edible wild mushrooms and single-cell proteins produced by fungi are also emerging as alternative protein sources due to their efficiency in converting waste materials into protein-rich biomass. Nevertheless, nucleic acid content, allergenic potential, other safety concerns, standardized production processes, and regulatory frameworks must be addressed [84]. Mycoprotein, derived from filamentous fungi, is another cost-effective and nutritionally beneficial alternative protein that serves as a sustainable meat substitute, providing essential nutrition acting as a prebiotic, antioxidant, and regulator of cholesterol, blood glucose, and muscle protein development [85,86]. Finally, microbial proteins produced using air show low land and water requirements reduced GHG emissions, and the potential to combat global malnutrition, aligning with global sustainability goals. For example, Solar Foods (a Finnish startup) produces a carbon-neutral microbial protein using CO_2_ from air, water, and minerals. The so-called “Solein” product offers versatile techno-functional properties in food applications, matching the properties of plant-based protein isolates. Its regulatory status varies by region, with the EU’s Novel Food status ensuring safety, while other areas have their regulatory processes, like GRAS in the USA [87]. Further research should comprehensively compare alternative proteins, considering different consumer segments to improve acceptance, influenced by attitudes, familiarity, taste, disgust, food neophobia, and social norms [88].

## 6. Food Processing and Food Technology

While the sustainability of the food industry relies on tried-and-true technologies, there are instances where these technologies become outdated. Thus, a key factor for success in food companies is the ability to continuously innovate, adapt to the ever-changing landscape, and maintain a competitive edge in the market. At present, one of the most disruptive manufacturing technologies in the food sector is 3D printing, which is approaching a global market worth nearly one billion dollars and has experienced substantial annual growth. This technology can revolutionize food texture design and generate tailored products with specific characteristics, flavors, colors, textures, and nutritional profiles. In addition, it empowers the customization of food based on individual preferences and needs, with applications ranging from space food and restaurant cuisine to distinctive 3D edibles, such as jellies, dough, pizza, pasta, biscuits, and chocolates [89].

Moreover, it involves multi-material printing and complex internal structures, enabling the development of healthier food products with reduced sugar, salt, and oil contents [90]. It addresses forthcoming food and environmental challenges, including integrating dried wheat worms into diets and diversifying flour [89]. However, further research is needed to optimize printing materials and parameters and to understand how material rheological properties impact processes such as extrusion [91]. Addressing the challenges related to consumer attitudes, awareness, standardization, and food material consistency regarding 3D-printed foods is essential, too [89].

Driven by the complexities of modern supply chains and consumer preferences for safe food, smart packaging (which includes active and intelligent technologies) is another innovation that becomes increasingly significant as it enhances the safety and quality of food products [92]. Active packaging incorporates elements aimed at preserving or extending the shelf life of products. At the same time, intelligent systems monitor packaged food conditions (e.g., temperature and pH) during storage and transportation without releasing any components into the food [93]. Due to strict regulations, Europe is currently behind in adopting active and intelligent packaging. However, the future food industry will be directed toward this path since smart packaging encompasses numerous benefits in safety, marketing, and logistics [94].

In addition, the demand for safer and more personalized food products and the current sustainability challenges have forced the food industry to reduce energy and resource consumption. Integrating non-thermal technologies into food processing is becoming increasingly important to maximize the conversion of raw materials into consumer products. Moreover, conventional processing and food separation techniques, like solvent extraction and membrane filtration, often reduce yields, diminish bioactive compounds’ content, and decline food quality [82,95]. Non-thermal technologies offer gentler processing conditions and food treatment without destroying their sensory and nutritional characteristics attributes while preserving the functionality of bioactive components during prolonged storage. These technologies include radio frequency drying, high-voltage electrical discharge, high-pressure processing, pulsed electric field processing, and microwave-assisted, ultrasound-assisted, and supercritical fluid extractions. Further research in the forthcoming years can address the potential limitations of these technologies, including their effects on bioactive lipids and interactions among food bioactives [82]. It is also necessary to reduce carbon footprints by adopting energy-efficient appliances, renewable energy sources, sustainable products, transportation practices, and agricultural methods [96].

## 7. Impact of Biotechnology

Crop improvement is essential for meeting the world’s increasing food, feed, and bioenergy demands. It has evolved from traditional breeding techniques (e.g., hybridization and selection) to proteomics and revolutionized biotechnological tools, integrating genetic engineering, genomics, and precision breeding that enhances productivity and adaptability to changing environments [97]. For example, environmental factors, such as drought, salinity, and biotic stresses, mainly related to climate change induce challenges to crop growth. Proteomics provides insights into plant molecular mechanisms, enabling the development of stress-resistant, high-yield crops through marker-assisted breeding by unraveling molecular mechanisms and revealing intricate proteomic behavior related to plant stress tolerance [98]. Moreover, by introducing targeted genes or employing advanced gene-editing tools, researchers can thoroughly alter crop characteristics, boosting them with resistance to pests, diseases, and environmental stresses [99].

To this end, the lustered, regularly interspaced short palindromic repeat (CRISPR)/CRISPR-associated protein (Cas) is a powerful genome editing tool that can be effectively used for precise genome editing of wheat and other crops. This system has revolutionized crop improvement with its simplicity and multiplexed gene editing capabilities, enabling rapid enhancements in less than a year compared to traditional methods that take 6–7 years [100]. Its applications in disease resistance, drought tolerance, and quality enhancement show promise for addressing food demands in the face of a growing population. In addition, integrating CRISPR/Cas9 with next-generation sequencing and advanced genomic methods enhances mutational screening and functional genomics, further propelling crop development [101]. Recent CRISPR/Cas applications in crops like Arabidopsis, rice, maize, soybean, and tobacco have improved yield and stress tolerance traits while avoiding stringent regulations and ethical concerns associated with permanent genetic modification. The challenges include designing accurate guide RNAs, selecting promoters, overcoming size limitations for viral packaging, and creating efficient, tissue culture-free editing methods [102]. CRISPR technology is also an emerging tool for ultra-sensitive pathogen and contaminant detection in packaged food, offering real-time surveillance and enhancing consumer health protection [103]. On the other hand, while genetically modified crops have the potential to improve yields, they also raise concerns regarding biosafety and environmental concerns, like altered crop behavior, herbicide and insecticide tolerance levels, transgene stacking, and biodiversity disruption. Ongoing research and informed decision making are vital for assessing their impacts and guiding their cultivation and use [104].

## 8. Cultural Diversity, Culinary Trends, and Sustainable Food Consumption

Globalization has fostered a global food culture, transcended borders, and promoted cultural appreciation through food. However, following some food scandals and conflicts related to genetically modified food production that have undermined public trust, consumers in the modern era are displaying a growing preference for traditional, locally sourced, organic, and slow food. The latter trend is expected to continue focusing on traditional, locally sourced, and sustainable food practices, appealing to consumers seeking a more authentic and ethical dining experience. Nevertheless, traditional food producers are continuously challenged to enhance the safety and convenience of their products to meet market demands while also grappling with the pressure induced by large retailers, open markets, and compliance with regulations. Subsequently, they expand their skills and adopt innovations to highlight their products’ nutritional benefits and health advantages. However, changing approaches risks altering the traditional appeal, which is met with consumer resistance, especially in the traditional food sector [105].

Driven by cultural diversity, culinary innovation plays a crucial role in this direction, encompassing the exploration of unique flavors and adapting seasonal variations and local ingredients. In addition, it facilitates the fusion of culinary traditions, emphasizing local ingredient sourcing, which not only preserves cultural traditions, but also aligns with sustainability goals, reducing carbon footprints and supporting local communities. Nonetheless, we should avoid assuming that local food automatically has a more negligible environmental impact than global food simply because it is produced closer to consumption [106]. For instance, restaurants are typical sources of environmental unsustainability that can occur either from wastages (food and non-biodegradable materials like plastics) and emissions (burning of fuels in the kitchen and vicarious sources like food sourcing and supply) [107]. Instead of simplifying food into “local” vs. “global” or “in season,” it is advisable to adopt a holistic perspective of the food system, recognizing the interplay between local and global factors among food actors, fostering methodological approaches addressing sustainability across all dimensions and facilitating tangible progress in sustainable food consumption [106]. To this end, traditional and indigenous food systems are inherently integrating sustainable practices, such as agroforestry and low-impact fishing [108]. Moreover, green and sustainable dining practices, such as farm-to-table concepts, zero-waste initiatives, and environmentally conscious menu choices, are continuously gaining popularity, satisfying consumer preferences and environmental objectives [109,110,111]. Sustainability practices in meal production, preparation, and service are vital to modern diners’ perception of “overall quality.” This trend entails services satisfying customers’ nutrition, hygiene, social, and cultural requirements [112]. Foodservice companies expected to grow include those specializing in food delivery and takeout services, driven by the increasing demand for convenience and the growth of online ordering platforms.

Additionally, health-focused and plant-based foodservice providers are likely to see growth as consumers prioritize healthier and sustainable dining options [3]. Opportunities also exist for local governments to implement policies that promote both human and planetary health, including measures to reduce food overconsumption, such as encouraging lower meat consumption and regulating ultra-processed foods [113]. Inclusivity in dining, addressing diverse dietary needs and cultural backgrounds, promotes a sense of belonging. Additionally, technological disruptions shape the future of dining experiences. For example, a proposed innovation involves a smart dining table that measures food weight to track consumed calories [114,115]. Fast food will likely emphasize healthier menu options, sustainability, and enhanced technology for ordering and delivery. Augmented and virtual reality innovative technologies offer immersive culinary adventures, influencing consumers’ behavioral, sensory, social, and intellectual perceptions. Therefore, restaurants can use augmented reality to create immersive experiences (e.g., through aesthetics and storytelling) that boost the overall food well-being of their customers, integrating their preferences [116]. Moreover, there are suggestions for a system that uses cameras to analyze food images, identify meal components and portion sizes, and estimate calorie intake through online databases [117].

## 9. Personalized Nutrition

Everything we consume is broken down into molecules that uniquely interact with our microbiome and genome. For example, probiotics can help with irritable bowel syndrome and prevent colorectal cancer. At the same time, vitamins A and C possess anti-carcinogenic properties, and folic acid prevents DNA mutations by enhancing protein methylation processes. Subsequently, health can rely on diets and micronutrients to reduce genetic mutations causing diseases [118]. In the present nutritional landscape, the traditional notion of “one-size-fits-all” dietary guidelines has given way to a more contemporary approach driven by individuals’ unique preferences. This transformation has led to the emergence of personalized nutrition, recognizing that each person is distinct and responds differently to the same foods [119]. Personalized nutrition follows the development of the nutrigenomics field that studies the relationship between nutrition and gene expression. Nutrigenomics explores more effective and customized approaches to diet based on the study of individual genetic and physiological variations influencing nutrient levels and dietary reactions, offering promising avenues for cancer prevention and the treatment of diseases [118,120].

Personalized nutrition integrates an individual’s dietary preferences and sustainability values into dietary recommendations, guiding them toward eco-friendly food choices [119]. In a world often facing obesity and related metabolic disorders such as type 2 diabetes and cardiovascular diseases, personalized nutritional recommendations offer promise for prevention strategies [121]. Challenges include the complex polygenic nature of nutrition-related diseases, necessitating the consideration of multiple genetic variants in predictive models and understanding gene–environment interactions, especially in diseases influenced by external factors [120]. Achieving this goal necessitates a collaboration between basic and clinical scientists and healthcare professionals to establish a comprehensive framework for implementing these discoveries on a population scale [121].

For food companies, personalized nutrition represents a significant avenue for innovation, enabling them to create tailored products and dietary solutions that align with consumers’ needs [119]. Nutrigenomics and deep phenotyping are just a few factors that must be considered to craft personalized and unbiased nutritional solutions [121]. Advanced “omics” technologies can also offer insights in this direction, but they require machine learning and other digital tools to improve health outcomes and reduce disease burdens [120]. For instance, implementing machine learning and artificial intelligence for predicting weight loss and health outcomes (e.g., through random forest models and neural networks) has the potential to enable the identification of factors affecting the prediction of an individual’s weight loss progress. This kind of model has the potential to assist and complement healthcare professionals in recommending tailored adjustments to energy intake and creating personalized nutrition plans [122]. Along this line, the potential of wearable and mobile chemical sensors to track and guide nutrition is expected to grow tremendously over the years to come [115]. Furthermore, various technologies, like microphones, piezoelectric sensors, and accelerometers, have been demonstrated to monitor actions such as biting, chewing, swallowing, and arm/wrist movements to assess calorie intake based on physical activity [123,124]. Integrating various sensor modalities (e.g., non-invasive wearable electrochemical ones) with data analysis techniques can bridge the gap between digital and biochemical analyses, and precision nutrition can be revolutionized, making personalized nutrition more effective [123].

## 10. Consumer Health and Wellness

The prevalence of chronic diseases as a leading global cause of death, associated with the overconsumption of processed foods and inadequate intake of plant-based foods, has led to a rise in health-conscious individuals adopting well-being strategies (e.g., the Mediterranean diet and increased physical activity). However, these approaches can be complex and impractical for many in contemporary societies. Consequently, many consumers have redirected their interest towards the active role of natural supplements. Over the last decades, there has been a considerable scientific focus on identifying the target active components in plant-based foods and healthy diets, exploring their potential anti-oxidative, anti-tumor, anti-inflammatory, anti-hyperlipidemic, anti-hypertensive, anti-microbial, and anti-viral activities, in addition to their essential nutritional functions [16].

Along this line, fortifying foods and boosting diets with nutritional components fostered innovation in the food industry ecosystem, leading to the widespread use of terms such as nutraceuticals [3,16]. Though lacking a universally accepted definition, nutraceuticals are medicinal agents possessing drug-like attributes capable of effectively addressing severe illnesses, like diabetes, atherosclerosis, cancer, neurodegenerative disorders, and hematological conditions. They harness the potential of bioactive compounds, such as tannins, polyphenols, flavonoids, alkaloids, and terpenoids, commonly found in healthy food products [125]. On the other hand, functional foods are typically recognized as products with proven advantages in enhancing specific physiological functions beyond fundamental nutrition, contributing to enhanced health and reduced disease risks [126]. Functional and custom-designed foods are created by incorporating specific ingredients into conventional or traditional food products and altering their properties through binding, structural changes, or interface modifications, all while imparting health-enhancing qualities to these products [82].

Food bioactives and nutraceuticals encounter challenges related to their intricate compositions and diverse modes of action, encompassing tasks like extraction, chemical characterization, assessing in vitro and in vivo bioactivity, and comprehending their interactions with organs and microbiota [127]. Their effectiveness depends on multiple factors, encompassing bioactivity, bioavailability, metabolomics, nutrigenomics, and their stability within the food matrix. For instance, when administered orally, bioactives face constraints imposed by gastrointestinal enzymes, acidic pH levels, the epithelial barrier, and the mucosal layer [82]. Despite concerns about efficacy, quality, clinical evidence, and self-medication risks, the demand for nutraceuticals and functional foods continues to grow, creating opportunities in the market, which is anticipated to flourish in the post-lockdown period, driven by the increased attention from health-conscious consumers [115]. Nevertheless, developing novel functional foods is multifaceted and financially demanding, with consumer adoption contingent upon numerous influencing factors. Comprehending consumer uncertainty and skepticism regarding these products is essential for effective product development, and a deep understanding of consumer preferences and attitudes is critical for businesses to adapt [126]. Functional foods allow consumers to improve their health while enjoying familiar and convenient dietary options. Still, it is crucial to ensure that the claims of health benefits are based on sound scientific evidence, and safety considerations should always be a priority [128].

## 11. Food Production within the Circular Bioeconomy

Food bioactives can be sourced from by-products generated during food processing. These materials are abundant in functional compounds like antioxidants (polyphenols and carotenoids), glucosinolates, proteins, and dietary fiber (pectin and β-glucan). At the same time, they can be re-used to fortify food products and cosmetics [9,82,129,130]. Moreover, the integral role of food waste valorization is pivotal in the emerging era, forming the foundation of the bioeconomy, which, in turn, constitutes the core of the circular economy. This circular approach aligns with the goals of climate-neutral economies aimed for by developed nations like Europe, the US, Japan, and China by 2050–2060 [131,132,133]. Likewise, utilizing inedible and unavoidable food waste and residues as biorefinery feedstock aligns with United Nations Sustainable Development Goal 12.3, targeting a reduction in food losses across the production and supply chain [134].

The transition of the current linear development model to a circular bioeconomy approach can enhance resilience by providing opportunities to valorize food waste and other biomass substrates. Achieving this goal involves addressing technical, economic, and scientific challenges through a multidisciplinary approach enabled by the biorefinery concept, which integrates processes to convert sources into various bio-based products [2,65,135]. Along these lines, pyrolysis and hydrothermal carbonization can convert biomass effectively into environmentally friendly biofuels, biochemicals, and valuable carbon-based materials [136]. Bioenergy is a precious product that can be utilized for electricity generation, heat production, and transportation, offering a sustainable solution that mitigates landfill-related issues and GHG gas emissions.

On the other hand, while food waste valorization is a promising field, posing challenges as a universal solution does not exist due to the varied nature of food waste and the necessity to ascertain feedstock composition and desired final products [137]. The efficient valorization of food waste within the bioeconomy requires addressing feedstock variability, technology selection, improving product efficiency, reducing production costs, and innovating separation methods [138]. Moreover, the primary aim is still to reduce food waste, and recovery approaches need to suit local conditions without disrupting the food supply or geopolitics. Efforts to enhance resource efficiency should account for the potential rebound effect, where greater efficiency can lead to increased resource consumption [139]. Future breakthroughs in food waste valorization and management can involve the metabolic engineering of microorganisms for product specificity [140]. Finally, an interdisciplinary collaboration is essential for cost-effective hybrid conversion platforms, with process models predicting the economic value to attract investments. However, scaling demands comprehensive product recovery and environmental impact assessments [137].

## 12. Conclusions and Future Perspectives

Achieving sustainable food production in the decades to come is a multifaceted challenge that requires comprehensive changes across various aspects of the food supply chain. Embracing crop rotations, organic farming, agroforestry, and vertical farming is crucial for optimizing agricultural productivity and resource management. Moreover, the future of food relies on innovative approaches that balance nutrition, health, and environmental responsibility. With innovations such as 3D printing, smart packaging, and non-thermal technologies, food processing technologies continue to evolve. The digitalization of the food supply chain through Industry 4.0 technologies offers excellent potential to address the challenges and enhance traceability, transparency, and food safety while optimizing supply chains, reducing food loss and waste, and promoting food security at the same time. Food security and climate-resilient supply chains are bound to alternative protein sources and the utilization of food processing by-products and waste that align with a climate-neutral, circular bioeconomy and sustainable development goals. Crop productivity and food security can be enhanced by using genome editing tools (e.g., CRISPR/Cas9). The ongoing research addresses the challenges related to nutritional completeness, consumer acceptance, safety, and regulations. Finally, the evolving food landscape embraces cultural diversity, culinary innovation, and sustainability trends, emphasizing traditional, locally sourced, and organic foods, sustainable dining practices, and personalized nutrition. In this ever-evolving food landscape, our collective commitment to innovation, sustainability, and the well-being of both people and the planet will shape a future where food inspires a positive change and resilience for future generations.

## Data Availability

Not applicable.
